# Radiofrequency ablation combined with conductive fluid-based dopants (saline normal and colloidal gold): computer modeling and ex vivo experiments

**DOI:** 10.1186/s12938-020-00842-8

**Published:** 2021-01-06

**Authors:** Dora Luz Castro-López, Enrique Berjano, Ricardo Romero-Mendez

**Affiliations:** 1 Facultad de Ingeniería, Universidad Autónoma de San Luis Potosí, San Luis Potosí, SLP 78290 México; 2 BioMIT, Department of Electronic Engineering, Universitat Politècnica de València, 46018 Valencia, Spain

**Keywords:** Gold nanoparticles, Nanofluid, Saline solution, Radiofrequency ablation

## Abstract

**Background:**

The volume of the coagulation zones created during radiofrequency ablation (RFA) is limited by the appearance of roll-off. Doping the tissue with conductive fluids, e.g., gold nanoparticles (AuNPs) could enlarge these zones by delaying roll-off. Our goal was to characterize the electrical conductivity of a substrate doped with AuNPs in a computer modeling study and ex vivo experiments to investigate their effect on coagulation zone volumes.

**Methods:**

The electrical conductivity of substrates doped with normal saline or AuNPs was assessed experimentally on agar phantoms. The computer models, built and solved on COMSOL Multiphysics, consisted of a cylindrical domain mimicking liver tissue and a spherical domain mimicking a doped zone with 2, 3 and 4 cm diameters. Ex vivo experiments were conducted on bovine liver fragments under three different conditions: non-doped tissue (ND Group), 2 mL of 0.9% NaCl (NaCl Group), and 2 mL of AuNPs 0.1 wt% (AuNPs Group).

**Results:**

The theoretical analysis showed that adding normal saline or colloidal gold in concentrations lower than 10% only modifies the electrical conductivity of the doped substrate with practically no change in the thermal characteristics. The computer results showed a relationship between doped zone size and electrode length regarding the created coagulation zone. There was good agreement between the ex vivo and computational results in terms of transverse diameter of the coagulation zone.

**Conclusions:**

Both the computer and ex vivo experiments showed that doping with AuNPs can enlarge the coagulation zone, especially the transverse diameter and hence enhance sphericity.

## Background

Radiofrequency (RF) ablation (RFA) is a minimally invasive procedure used to thermally destroy tumors [1]. During the procedure a needle-like ablation electrode is inserted into the tumor and electrical current (~ 500 kHz) is conducted between this electrode and a large-surface dispersive electrode placed on the patient’s skin [2]. The electrical power is converted into heat by the Joule effect and causes cell death by coagulative necrosis when tissue temperature exceeds 50 °C for several minutes [3]. The thermally damaged tissue is known as the *coagulation zone* [4]. The therapeutic goal is to achieve a coagulation zone that covers the entire tumor. Unfortunately, the coagulation zone size is strongly limited by the appearance of a phenomenon called *roll-off*, which consists of the cessation of RF power due to a sudden increase in electrical impedance when the active electrode is completely surrounded by desiccated tissue (i.e., at ~ 100 °C) [5].

Larger coagulation zones could possibly be achieved by delaying *roll-off* as long as possible. Past studies have suggested injecting conductive fluids (such as saline solutions) into the target site before and/or during RFA [6–8]. The idea behind this ‘fluid-modulated RFA’ is to increase the electrical conductivity of the fluid-doped tissue and hence increase the deposited RF power in the target site. In theory, there is a direct relationship between saline concentration in the doped sample and its electrical conductivity *σ* [9]. In practical terms, the higher *σ* can delay the appearance of the first roll-off [10], which is crucial, since the coagulation zone volume is not greatly affected by power reapplications after this event [11] and increasing the saline infusion volume does not always lead to larger coagulation zones [12].

Since better results can be obtained by increasing dopant conductivity [8], nanofluid (NF), i.e., a fluid based on metallic nanoparticles (NPs) appears to be a suitable option. Most of the studies that combined NPs and RFA were not in the context of invasive RFA (where an electrode is inserted into the tumor), but rather in noninvasive RF-induced localized hyperthermia, in which the tumor is doped with NPs and RF fields are externally generated [13, 14]. In fact, very few studies that combine NPs with invasive RFA have been published [15–18]. Merkle et al.[15] studied the effect of doping the medium with superparamagnetic iron oxide prior to RFA in agar phantoms and in vivo liver models and found no significant differences in terms of coagulation zone size in the in vivo model, possibly because the NF containing iron oxide was administered intravenously, which did not ensure effective doping of the target area. In contrast, Pedro et al. [16] did find larger coagulation zones when RFA was combined with intravenous administration of colloidal gold NF in an in vivo model (VX2 tumor in kidney). Wu et al. [17] studied the effect of injecting carbon-coated iron NF during RFA in an ex vivo model and found that this resulted in larger coagulation zones than those obtained by injecting saline only. In this case, the NF was injected directly through the electrode to ensure the doping of the target area. Likewise, Jelbuldina et al. [18] studied the effect of injecting NF based on ferromagnetic particles prior to RFA in an ex vivo liver model and reported significantly larger coagulation zone sizes.

Although these experimental results seem promising so far, there is still little information on the effect of NF injection prior to RFA on the size of the coagulation zone and its comparative advantage over saline injection. To fill this gap, we conducted a study with two objectives: (1) to characterize the changes in electrical conductivity of a substrate doped with either 0.9% NaCl (normal saline) or 0.01 wt% colloidal gold (colloidal suspension of gold NPs–AuNPs in deionized water), and (2) to carry out a computer modeling study and ex vivo experiments on the effect of both dopants on the coagulation zone volumes created during RFA. While the numerical model provided information on the effect of RFA on different dopant concentrations and doped zone size, the ex vivo experiments were performed with a reduced set of parameters to verify the conclusions of the simulations. The results of this investigation revealed the advantages of using fluid-based dopants to produce larger tissue coagulation zones by delaying the first roll-off and suggest the appropriate tumor size and applied voltage conditions under which these effects can be produced.

## Results

### Electrical characterization of doped phantoms

Table 1 shows the results of the impedance and electrical conductivities measured in the agar phantom samples. As expected, the single agar samples had the lowest conductivity, which increased after doping the sphere with NaCl or AuNPs solution. The highest conductivity was obtained for 1.5 wt% of NaCl solution (0.145 S/m), followed by 1 wt% of AuNPs solution (0.138 S/m) and 1.0 wt% of NaCl solution (0.113 S/m). The Analysis of variance confirmed that significant differences in the *Z* values between the groups. The increase in electrical conductivity of the substrate agar gel can be seen in the results in Table 1. Bennett [9] experimentally found the following frequency-independent (up to 100 kHz) linear relation between NaCl concentration and electrical conductivity of agar phantoms doped with NaCl:

1 $$ {\sigma  }\left( {{\text{S}}/{\text{m}}} \right) = 215 \times \frac{{\left( {{\text{grams}}\;{\text{of}}\;{\text{NaCl}}} \right)}}{{\left( {{\text{solution}}\;{\text{volume}},\;{\text{mL}}} \right)}} + 0.0529. $$

**Table 1:** Impedance measured from phantom samples (*n* = 10) and *σ* values (estimated from computer simulations) of the agar-gel cylinder and of sphere

Phantom sample	*Z* (Ω)	*σ*_agar-gel_ (S/m)	*σ*_sphere_ (S/m)
Agar	322.4 ± 2.7	0.067	0.067
Agar + NaCl (1 wt%)	213.6 ± 2.4	0.067	0.113
Agar + NaCl (1.5 wt%)	177.5 ± 4.1	0.067	0.145
Agar + AuNPs (1 wt%)	185.9 ± 6.2	0.067	0.138

The residual value we found without NaCl (0.067 S/m) is more or less in agreement with the offset reported by Bennett [9] in Eq. (1) and is possibly associated with the insoluble component of the agar phantoms at room temperature since deionized water has extremely low electrical conductivity, < 0.2 mS/m. When Eq. (1) was used to estimate the electrical conductivities in our cases of NaCl doping, we obtained smaller values than those found experimentally (e.g., 0.09 S/m instead of 0.113 S/m for NaCl 1 wt%; and 0.1 S/m instead of 0.145 S/m for NaCl 1.5 wt%). This disagreement could be due to the accumulated errors in the experimental measurement of *Z* and the subsequent estimation of *σ* by computer modeling. We thus proposed a similar equation to Eq. (1) that relates the electrical conductivity of a substrate doped with 0.01% AuNPs with its concentration in the substrate. Based on the experimental data obtained (1 wt%, 0.138 S/m) this would be:

2 $$ \sigma \left( {{\text{S}}/{\text{m}}} \right) = 0.071\cdot\left( {wt\% } \right) + \sigma_{S} , $$

where *σ*_*S*_ is the electrical conductivity of non-doped substrate. It should be emphasized that this expression is only approximate, since it is based on a single concentration value. Theoretical estimates of the electrical conductivity of AuNPs colloidal solutions or substrates doped with this solution are difficult to calculate, since they depend on many factors, including the measurement frequency or NP size [19]. In this respect, expression (2) would be limited to a frequency of 500 kHz and NPs size of 10 nm.

### Computational results

**Fig. 1:**
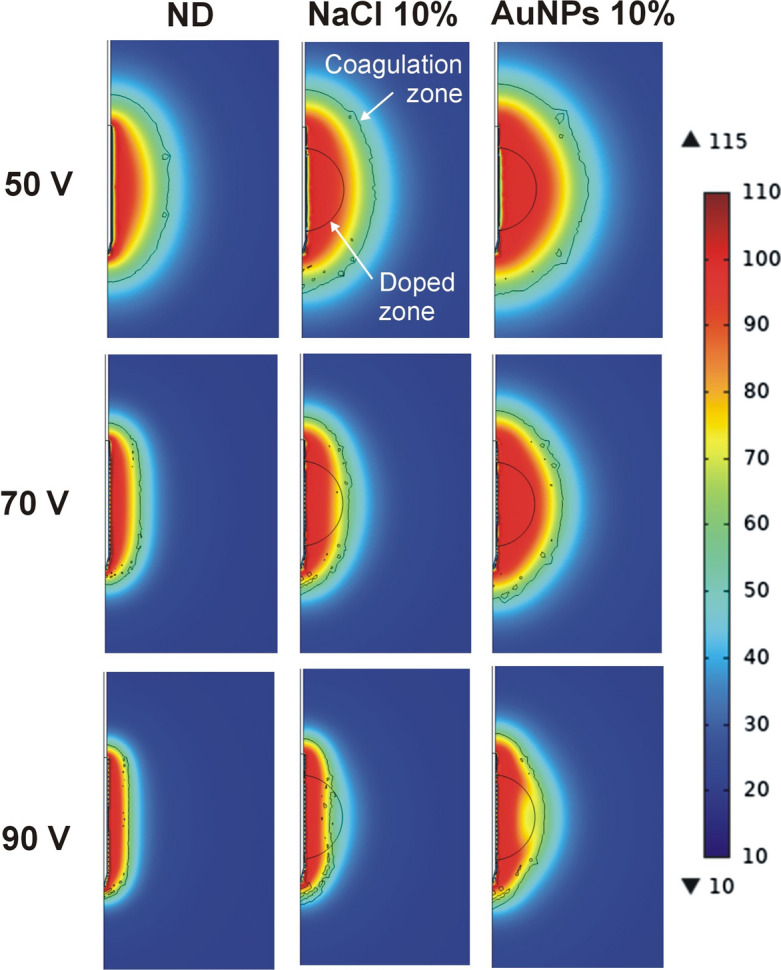
Temperature distributions computed at roll-off time for different values of applied voltage on a 2-cm spherical zone doped with NaCl and AuNPs solutions at 10%. ND: non-doped tissue case (scale in °C)

As the results showed that the doped zone has a crucial effect on the temperature distributions and the size of the coagulation zone, we give the results for each doped zone size separately. Table 2 shows the results for 50, 70 and 90 V RFA until roll-off for the 2-cm doped zone, and Fig. 1 gives the related temperature distributions. As expected, initial impedance declined slightly as dopant concentration increased, with generally lower values in the case of AuNPs. Time to roll-off (time to reach 100 Ω, t_100-Ω_) showed relatively similar values at 50 V (450 − 479 s), with the highest value for AuNPs at 10%. The delay in roll-off implied a longer ablation time and hence a greater amount of delivered energy. In terms of coagulation zone size, transverse coagulation diameter values increased with dopant concentration, from 2.6 cm for ND to 4.4 cm for AuNPs at 10% and in this case the coagulation volume tended to be more spherical (see Fig. 1). When applied voltage was increased to 70 and 90 V, roll-off occurred earlier and coagulation zones were smaller, especially due to the reduced transverse diameter, since the axial diameter was almost unaffected by the applied voltage.

**Table 2:** Results of the RFA for different values of applied voltage on a 2-cm spherical zone in case of non-doped tissue (ND) and two types of dopants: saline solution (0.9% NaCl) and AuNPs (0.01 wt%)

Dopant	*Z*_i_ (Ω)	50 V			70 V			90 V		
		*t*_100-Ω_	*B*	*A*	*t*_100-Ω_	*B*	*A*	*t*_100-Ω_	*B*	*A*
ND	87.7	457	2.7	4.4	94.5	1.6	3.8	31.5	1.0	3.7
NaCl										
1%	84.6	454	2.8	4.3	95	1.7	4.0	35	1.2	3.7
2%	82	452	2.8	4.3	97.5	1.7	4.0	35.5	1.2	3.7
5%	75.5	457	3.1	4.6	103	1.9	4.0	36	1.3	3.8
10%	67.8	450	3.5	5.0	109.5	2.2	4.2	37.5	1.3	3.9
AuNPs										
1%	78	456	3.0	4.5	101	1.8	4.0	35.5	1.2	3.6
2%	71.5	451	3.2	4.7	106.5	2.0	4.1	36.5	1.3	3.8
5%	60	448	3.7	4.8	120.5	2.6	4.2	41	1.5	4.0
10%	51.6	479	4.4	5.0	144	3.2	4.4	49.5	2.3	4.0

*Z*_i_: initial impedance; *t*_100-Ω_: time until roll-off (in s); B and A: transverse and axial diameters (in cm) of the coagulation zone, respectively. The different values of concentration (from 1 to 10% are volume fraction)

**Fig. 2:**
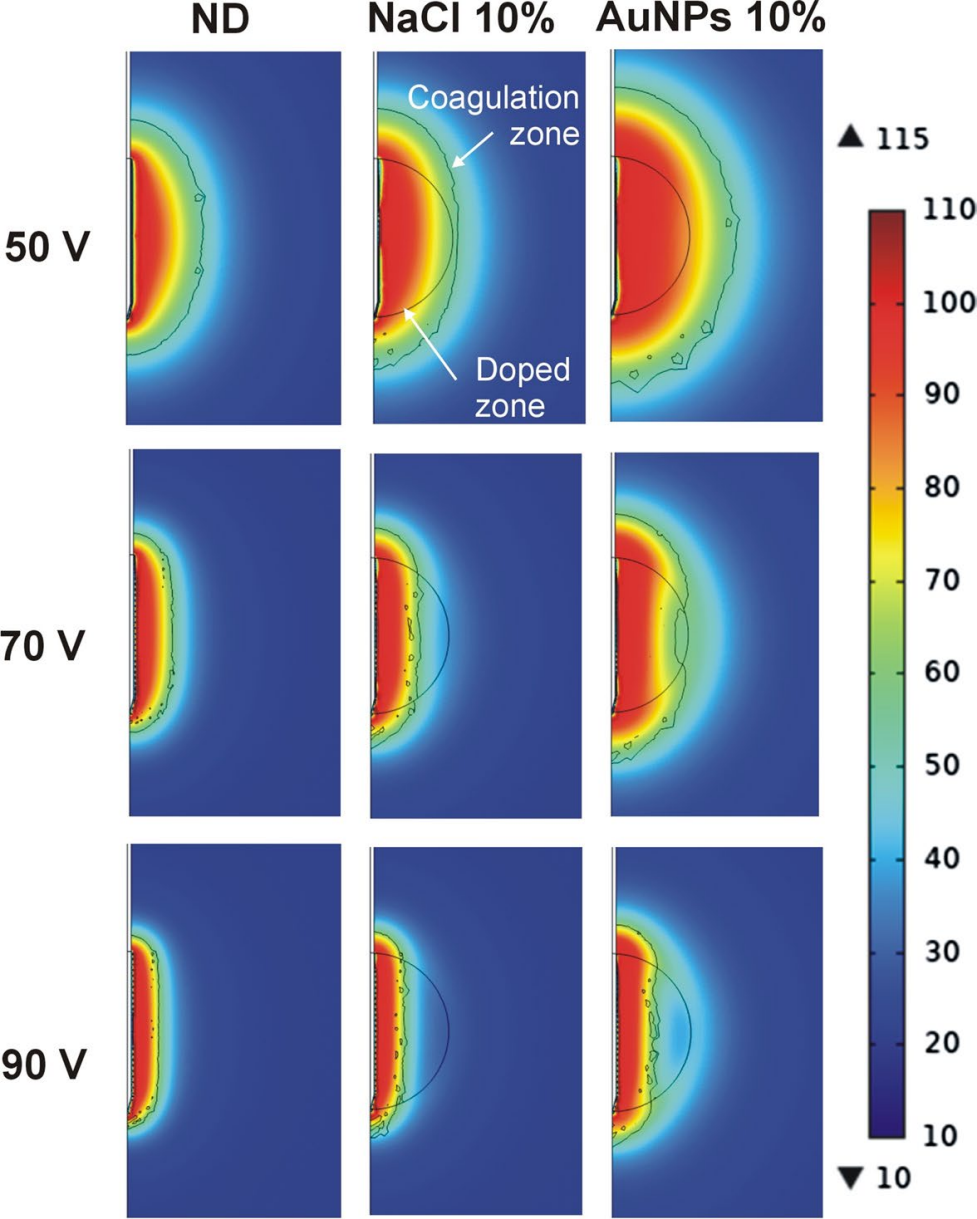
Temperature distributions computed at roll-off time for different values of applied voltage on a 3-cm spherical zone doped with NaCl and AuNPs solutions at 10%. ND: non-doped tissue case (scale in °C)

**Fig. 3:**
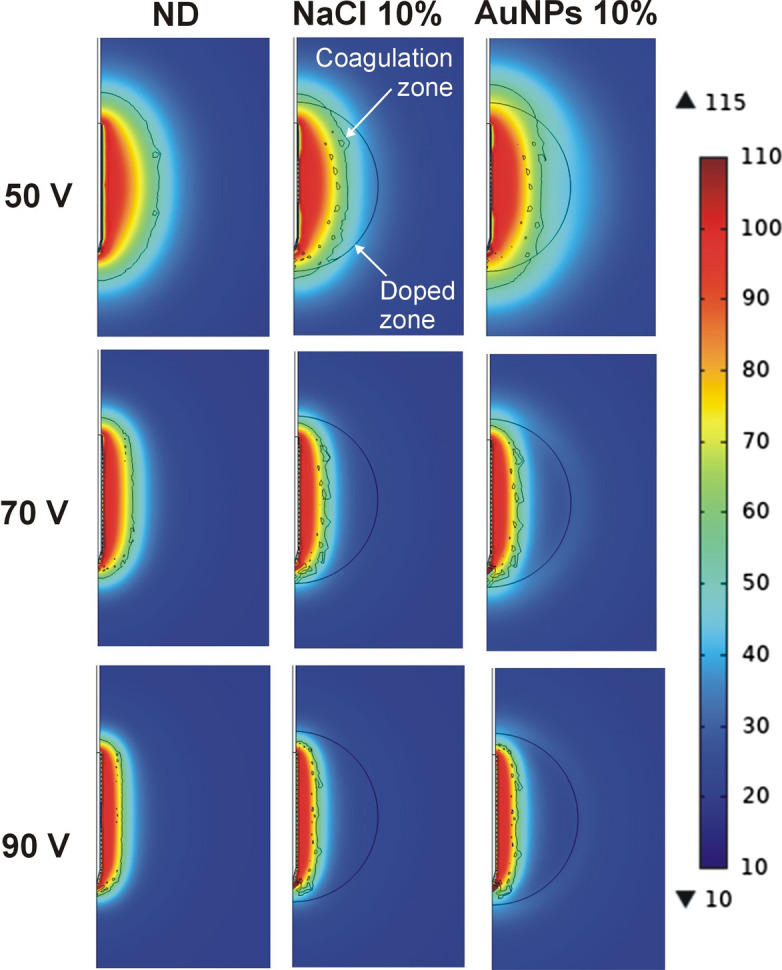
Temperature distributions computed at roll-off time for different values of applied voltage on a 4-cm spherical zone doped with NaCl and AuNPs solutions at 10%. ND: non-doped tissue case (scale in °C)

Table 3 shows the results of the 3-cm doped zone for 50, 70 and 90 V RFA up to roll-off, with the associated temperature distributions in Fig. 2. Initial impedance declined as dopant concentration increased and reached lower values than the 2-cm doped zone, with shorter roll-off times. Table 4 shows the results of the 4-cm doped zone at 50, 70 and 90 V RFA up to roll-off, with the associated temperature distributions in Fig. 3. Initial impedance was even lower than in the 3-cm doped zone and declined as dopant concentration increased. Times to roll-off were even shorter than in the 3-cm doped zone and the axial and transverse coagulation diameters were smaller, especially for 50 V. Overall, we observed that the coagulation zone diameter decreased as the diameter of the doping zone increased.

**Table 3:** Results of the RFA for different values of applied voltage on a 3-cm spherical zone in case of non-doped tissue (ND) and two types of dopants: saline solution (0.9% NaCl) and AuNPs (0.01 wt%)

Dopant	*Z*_i_ (Ω)	50 V			70 V			90 V		
		*t*_100-Ω_	*B*	*A*	*t*_100-Ω_	*B*	*A*	*t*_100-Ω_	*B*	*A*
ND	87.7	465.5	2.7	4.4	94.5	1.6	3.8	31.5	1.0	3.7
NaCl										
1%	82.5	438	2.6	4.4	92	1.8	3.9	32	1.2	3.8
2%	78	421.5	2.7	4.5	90	1.8	4.0	31.5	1.2	3.8
5%	67.7	392	2.7	4.6	86.5	1.8	4.2	30	1.2	3.8
10%	57	382	3.1	4.8	87	1.8	4.2	30.5	1.3	3.9
AuNPs										
1%	71.7	402	2.7	4.5	88	1.8	4.1	30.5	1.3	3.7
2%	62	385	3.0	4.8	86.5	1.8	4.2	30	1.3	3.8
5%	47.6	399.5	3.0	4.8	97.8	1.9	4.3	34	1.3	4.0
10%	38	468	4.5	5.5	126.5	2.5	4.8	43.5	1.7	4.2

*Z*_i_: initial impedance; *t*_100-Ω_: time until roll-off (in s); *B* and *A*: transverse and axial diameters (in cm) of the coagulation zone, respectively. The different values of concentration (from 1 to 10% are volume fraction)

**Table 4:** Results of the RFA for different values of applied voltage on a 4-cm spherical zone in case of non-doped tissue (ND) and two types of dopants: saline solution (0.9% NaCl) and AuNPs (0.01 wt%)

Dopant	*Z*_i_ (Ω)	50 V			70 V			90 V		
		*t*_100-Ω_	B	A	*t*_100-Ω_	*B*	*A*	t_100-Ω_	*B*	*A*
ND	87.7	440	2.7	4.4	94.5	1.6	3.8	31.5	1.0	3.7
NaCl										
1%	81.4	400	2.7	4.4	87	1.6	3.9	30	1.2	3.6
2%	76	367	2.6	4.4	80	1.7	3.9	29	1.2	3.6
5%	64	306	2.5	4.3	66	1.7	3.9	25.5	1.2	3.6
10%	52	269	2.5	4.4	55	1.7	3.8	22.5	1.2	3.4
AuNPs										
1%	69	327	2.5	4.4	71	1.7	3.9	27	1.2	3.6
2%	58	287	2.5	4.4	60	1.7	3.9	23.5	1.2	3.6
5%	42	245	2.5	4.4	50.5	1.6	3.8	20.5	1.2	3.6
10%	31.9	265	2.6	4.9	54.5	1.7	3.9	21.5	1.2	3.6

*Z*_i_: initial impedance; *t*_100-Ω_: time until roll-off (in s); *B* and *A*: transverse and axial diameters (in cm) of the coagulation zone, respectively. The different values of concentration (from 1 to 10% are volume fraction)

### Ex vivo results

**Fig. 4:**
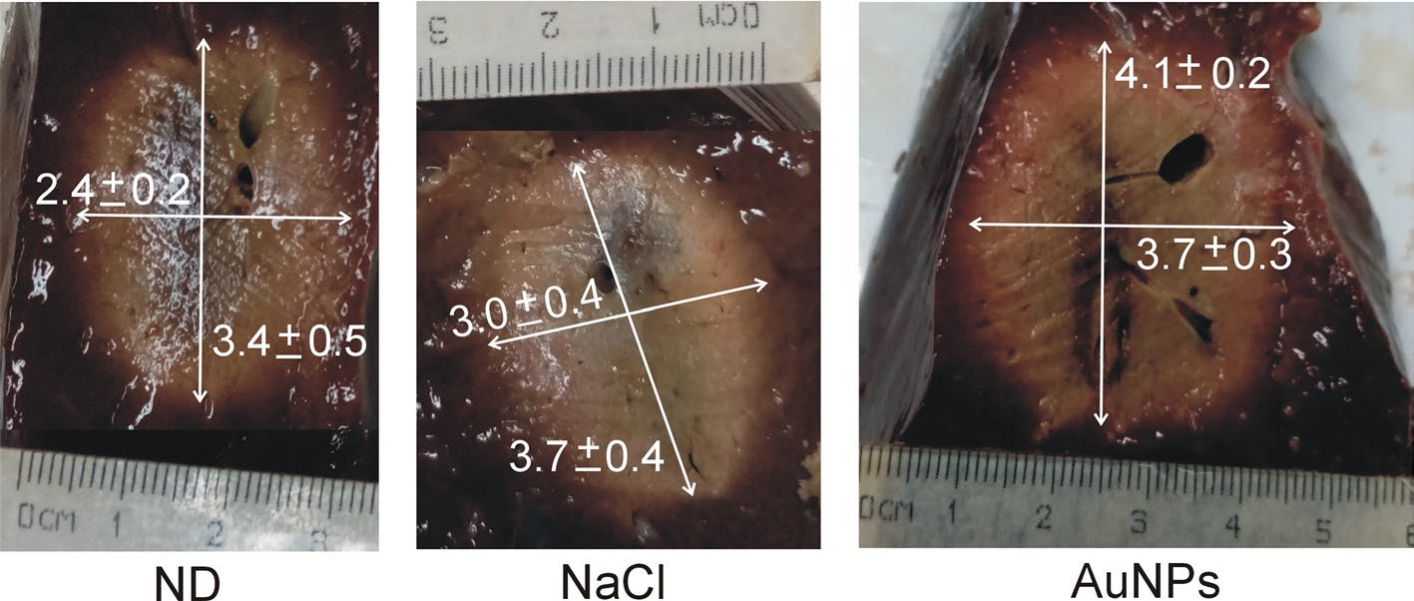
Examples of coagulations created with RFA on non-doped (ND) liver samples and on samples doped with 2 mL of 0.9% NaCl (NaCl) and 2 mL of 0.1 wt% AuNPs. Constant voltage of ~ 57 V was applied until roll-off (scale in mm, measurements in cm)

Table 5 gives the results of the ex vivo experiments and Fig. 4 shows examples of the coagulations for each doping condition group up to roll-off, which occurred earlier for the ND (281 ± 31 s) than for AuNPs Group (432 ± 36 s). There was a direct relationship between time to roll-off (*t*_100-Ω_) and coagulation zone size, with the smallest diameters in the ND Group (2.4 ± 0.2 cm) and the largest in the AuNPs Group (3.7 ± 0.3 cm). There were no significant differences in the initial impedance of the groups: 70.7 ± 6.3 Ω, 73.2 ± 4.0 Ω and 74.4 ± 2.4 Ω, for ND, NaCl and AuNPs Groups, respectively, which could be due to the low precision of the RF generator (± 10%). When the coagulation zone reached the outer tissue surface, the transverse diameter was assessed by the radius of the deepest zone (i.e., not in contact with the surface).

**Table 5:** Results of the ex vivo experiments for the three considered groups (*n* = 4, mean ± standard deviation)

Group	Coagulation diameters		*t*_roll-off_ (s)
	Transverse (cm)	Axial (cm)	
ND	2.4 ± 0.2	3.4 ± 0.5	281 ± 31
NaCl	3.0 ± 0.4	3.7 ± 0.4	379 ± 12
AuNPs	3.7 ± 0.3	4.1 ± 0.2	432 ± 36

*t*_roll-off_: time until roll-off (at which point the ablation ceased). ND Group: non-doped tissue; NaCl Group: infusion of 2 mL of 0.9% NaCl; AuNPs Group: infusion of 2 mL of 0.1 wt%. AuNPs

We also conducted computer simulations mimicking the applied voltage used during the ex vivo experiments (57 V). When a 2-cm doped zone was assumed, the transverse diameter of the coagulation zone was 2.2 cm for ND, while it ranged from 2.4 to 2.9 cm for NaCl, and from 2.6 to 3.8 cm for AuNPs (for concentrations of both dopants varying from 1 to 10%). These values were very similar to those obtained in the experiments. In contrast, the times to roll-off predicted by the computer model (222 − 289 s) were in general shorter than those measured in the ex vivo experiments (281 − 432 s). When larger doped zones were considered (3- and 4-cm diameters) the model predicted smaller transverse diameters than those obtained in the ex vivo experiments.

## Discussion

The first step was to assess how the properties of the doped substrate changed with dopant type and concentration. Two fluid-based dopants were considered, normal saline and colloidal gold. While saline infusion has been clinically used at different concentrations to dope RFA target tissue [12, 20], colloidal gold is little used in clinical practice [21]. The use of a fluid-based dopant before and during RFA is associated with the following two phenomena: (1) higher substrate electrical conductivity, especially if hypertonic saline (> 3%) is used instead of normal saline (0.9%); and (2) roll-off is delayed due to rehydration of the desiccated tissue (only with continuous infusion or periodic administration of bolus during RFA [20]). Only the first of these was considered in the present study, since we assumed that doping was by infusion before RFA (i.e., by pre-injection), in which case the dopant is only expected to alter the substrate properties.

Although some authors [22] have suggested that NPs could significantly raise the substrate thermal conductivity by up to ~ 23%, this would only be true at very high concentrations (e.g., 4% volume fraction [22]). In fact, our theoretical estimates (see Table 6) showed that the volume occupied by the NPs is much smaller, e.g., 5 × 10^−5^% when 10% colloidal gold (0.01 wt%) is infused into the substrate. Our estimates (see “Computer modeling of RFA in a doped zone” section) therefore showed that only the electrical conductivity is substantially modified by the effect of saline and colloidal gold-based dopants, at least at the concentrations considered here (less than 10 wt%). This is in agreement with previous estimates of the properties of saline-mixed tissue, when electrical conductivity was seen to change drastically with the saline:tissue mixing ratio while the thermal conductivity hardly changed [23].

**Table 6:** Estimation of the characteristics of the tissue doped with different concentrations (*C*, volume fraction) of a solution of 0.01% (wt) AuNPs and a solution 0.9% NaCl

Dopant	*C* (%)	*Φ*_D_ (× 10^−9^)	*σ* (S/m)	*c* (J/kg K)	*k* (W/m K)
	0 (non-doped)	0	0.2	3455	0.502
0.9% NaCl	1	−	0.219	3455	0.502
	2	−	0.238	3455	0.502
	5	−	0.297	3455	0.502
	10	−	0.394	3455	0.502
0.01 wt% AuNPs	1	54.15	0.271	3455	0.502
	2	108.30	0.342	3455	0.502
	5	270.75	0.555	3455	0.502
	10	541.50	0.910	3455	0.502

*σ:* electrical conductivity, *c*: specific heat, *k:* thermal conductivity, tissue properties estimated at 37 °C. In the case of AuNPs *σ* was estimated using the *Φ*_D_ value (volume fraction of solid AuNPs within the doped tissue); while that in the case of NaCl *σ* was estimated using Eq. (1) proposed in [9]

Although the electrical properties of colloidal gold have previously been evaluated [24], no study has so far evaluated the electrical conductivity of AuNPs–substrate mixtures. Our data, shown in Table 6, provide guiding values on how the electrical conductivity of a substrate doped with colloidal gold could change at different weight concentrations between 1 and 10%. It is reasonable to assume that a higher concentration of doping agent in the substrate (> 10%) and other more conductive types of dopants (e.g., hypertonic saline or > 0.01 wt% colloidal gold) would provide higher electrical conductivity values.

Once it was found that the addition of nanofluid (normal saline or colloidal gold 0.01 wt%) to a substrate at concentrations between 1 and 10% only modified its electrical conductivity, we explored how this could alter the electrical and thermal performance of a spherical doped zone during RFA. For this, we built a numerical model based on a doped zone surrounded by non-doped tissue, i.e., similar to a two-compartment model, as proposed in [25, 26]. These models have already shown that the presence of a tumor with higher electrical conductivity than the surrounding tissue provides very different temperature distributions than models based on homogeneous tissue (one-compartment models) [25, 26]. They also suggested that the maximum voltage applied before roll-off is different for different sized tumors and reduces as tumor diameter increases [26]. None of the previous models considered different tissue doping conditions or assessed the effect of doped zone size in relation to electrode length.

Our computational results showed that in the non-doped (ND) tissue model the relationship between doped zone diameter and electrode length determines RFA electrical and thermal performance. As the doped zone size exceeds the electrode length (i.e., the electrode is completely inside the doped zone, as in the 4-cm case), the temperature distribution is more similar to the homogeneous tissue RFA case, i.e., electrical power deposition (or current density) and heating mainly occur at the edges of the electrode (see Fig. 3). This is known as the *edge effect* and greatly limits the growth of the transverse diameter and sphericity of the coagulation zone.

On the other hand, we also found that if the electrode length exceeds the doped zone diameter and the edges of the electrode are outside the doped zone (as in the 2-cm case), the *edge effect* is in some way compensated for by the high electrical conductivity of the doped zone, which means that the current density is higher in the central zone than at the edges, where independent heating zones can be seen in the first few seconds (see Fig. 1). This behavior was amplified by the presence of a highly conductive dopant, insofar as the current density was greater than in the non-doped tissue case. As a result, the computational results shown in Figs. 1, 2, 3 suggest that the following procedure would be an effective way of ablating a spherical tumor: (1) electrode longer than the tumor diameter (e.g., 1 cm longer), (2) tumor in the central area of the electrode, i.e., distal and proximal edges surrounded by healthy tissue, and (3) tumor doped with a sufficiently high concentration of conductive agents (for example, 10% AuNPs 0.01 wt%). This would create relatively spherical coagulation zones capable of destroying the tumor plus a safety margin (see Fig. 1). From an oncological point of view, we recognize that traversing the tumor with the sharp tip of the electrode and exceeding its limits would imply a clear risk of needle track seeding prior to RFA (i.e., tumor cell spread [27]). However, it is also true that the tissue adjacent to the tip will most certainly be ablated, greatly reducing this risk.

Our results also suggest that when RFA is performed on highly conductive substrates (note that this may be valid both for tumors doped with a conductive substance and for non-doped tumors with much higher conductivity than the surrounding tissue), larger coagulation zone volumes can be created at low voltages (50 V). However, moderate and high voltages (70 and 90 V) can quickly heat the tissue but involve the risk of early roll-off, which notably limits the growth of the coagulation zone transverse diameter (see Figs. 1, 2, 3). This fast RFA heating on a highly conductive substrate was demonstrated by Ji et al.[28] in an ex vivo study in which the evolution of tissue temperature at 1 and 2 cm from the electrode was recorded. Both our computer and experimental results (see Table 1 and Fig. 4) suggest that when the electrical conductivity of the dopant is raised, the transverse diameter is larger than the axial diameter, so that the coagulation zone is more spherical, which is in agreement with Ji et al.*’*s results [28]. However, their experimental setup and ours share the uncertainty about the exact distribution of the dopant around the electrode. For this reason, our computer results suggest that in both experimental models the dopant was possibly concentrated in the central electrode zone, thus achieving preferential heating in that zone, plus a more spherical coagulation zone.

Our conclusion on the recommended use of low voltage instead of high voltage goes in the opposite direction to the impedance-controlled pulsed protocol, which employs high-voltage pulses and is broadly used in clinical practice. This protocol was demonstrated to be better than a low-voltage continuous protocol in a classical work by Goldberg et al.[29], later improved on by Solazzo et al.[30]. However, these studies used homogenous tissue, i.e., a non-doped target. Other experimental studies on RFA combined with saline infusion could deliver RF power at a high voltage with few roll-offs [31]. In fact, our clinical experience of ablating tumors smaller than 2 cm with a 3-cm electrode with hypertonic saline infusion only in the central electrode zone showed that it was possible to deliver power without roll-offs when an extra bolus is infused after 4 min [20]. The discrepancy of these results with those from our model is that the dopant fluid possibly provides an extra hydration effect which could be the partial cause of the roll-off delay, as we modeled in [32]. Our computer results call attention to the need to explore optimized protocols for the case of substrates doped with highly conductive fluids and suggest that in these cases the relationship between electrode length and tumor size could condition the result in terms of coagulation zone volume.

Our computer and ex vivo results showed good agreement in terms of coagulation zone transverse diameter when we assumed a 2-cm-diameter doped zone, which suggests that during *ex viv*o experiments the dopant possibly extended in an area of that size. The times until roll-off predicted by the computer model were shorter than those observed during the ex vivo experiments. This could be due to the fact that the computer model did not include the possible rehydration effect of the doping fluid, which has been shown to slightly delay the appearance of the roll-off [32].

Finally, although there are still no data available on the advantages of nanofluids over saline to dope targets, it should not be forgotten that there are serious risks associated with infusing large amounts of fluid in RFA [33], and it seems reasonable to suggest that higher electrical conductivity should be achieved with the smallest possible amount of fluid.

### Limitations

Some limitations should be pointed out. First, our theoretical estimations showed that the presence of either NaCl or AuNPs at the concentrations considered only affected the electrical characteristics of the substrate. This might not be valid with fluids at higher concentrations (i.e., > 0.9% in case of NaCl, and > 0.01 wt% in case of colloidal gold) or when the substrate is doped with higher concentrations of dopant fluid (i.e., > 10%). It is also important to point out that the theoretical estimates of density, volumetric heat specific and thermal conductivity of the doped substrate were based on expressions that have been proposed to study tissues with variable water content [34]. Second, our modeling study assumed that the dopant was spherically distributed around the electrode. This should be seen as a first approximation to the real situation, which could be different in the case of a heterogeneous tissue in which blood vessels could preferentially evacuate the dopant agent [35]. Third, our model considered that electrical conductivity of doped substrates dropped 2 orders of magnitude once temperature reached 100 ºC. However, there are no experimental data available on electrical conduction through desiccated tissues previously doped with AuNPs, i.e., we do not yet know if the ‘dry residue’ formed by the NPs themselves can conduct the RF current in any way. And fourth, Eqs. (1) and (2) which relate the electrical conductivity (*σ*) of the doped substrate with the dopant concentration (AuNP or NaCl) were really built from very little experimental data, which also have an associated uncertainty. Furthermore, we are also not completely sure that the relationships are necessarily linear. In this regard, future experimental studies should be carried out to establish more accurately the relationship between dopant concentrations and the resulting electrical conductivity, especially under conditions of perfusion of blood (in vivo) and tumor tissue. As a consequence, since our study did not assess the impact of these limitations by means of sensitivity analyses, we recognize that our conclusions could be different in the case of being able to have more exact relationships between *σ* and dopant concentration.

## Conclusions

The theoretical analysis showed that the addition of normal saline or colloidal gold (0.01 wt%) at concentrations lower than 10% only modify the electrical conductivity of the doped substrate and have very little effect on the thermal characteristics. The computer results showed a relationship between doped zone size and electrode length regarding the created coagulation zone, and that highly conductive doped substrates possibly require low voltages to obtain large spherical coagulation zones. Both computer and ex vivo experiments showed that doping with AuNPs can enlarge the coagulation zone, especially the transverse diameter, hence achieving more spherical coagulation zones. These findings indicate that fluid-based dopants produce larger coagulation zones by delaying the first roll-off, which suggests that combining RFA with tumor doping could improve current ablation techniques by achieving complete coagulation of the tumor zone with a single application, i.e., without overlap due to repositioning of the electrodes.

## Methods

### Electrical characterization of the doped substrates

**Fig. 5:**
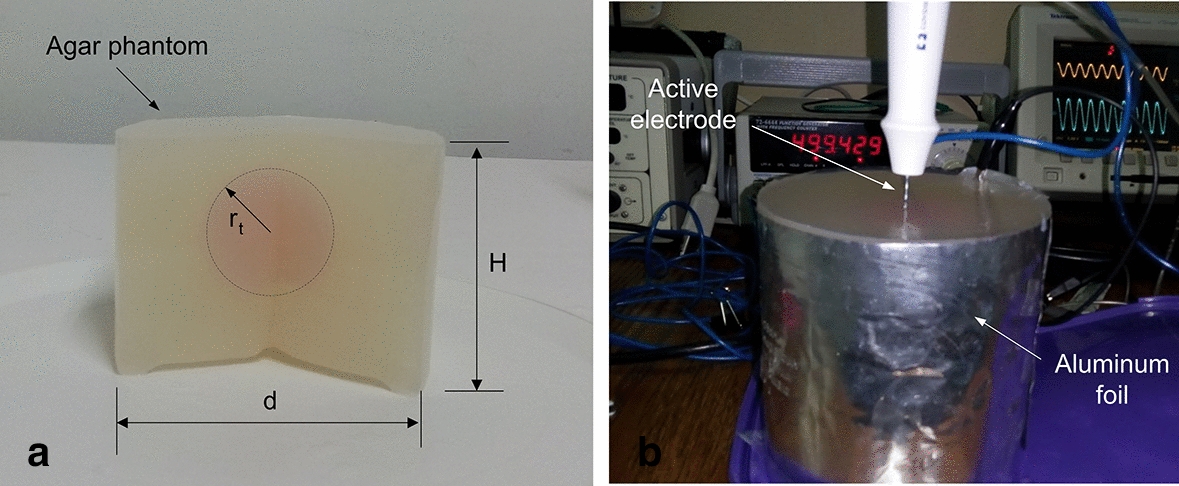
**a** Agar phantoms were based on a sphere of radius *r*_t_ = 2 cm (dotted line) located at the center of a cylinder of diameter *d* = 10 cm and height H = 11 cm. Note that a circular fragment of the cylinder has been removed to facilitate the observation of the central sphere. **b** Experimental setup used to electrically characterize each phantom, including an RF applicator (active electrode) inserted into the center of the sphere doped with NaCl or AuNPs. The entire phantom was surrounded by a 2-mm-thick aluminum foil that acted as a dispersive electrode

In order to quantify changes in electrical conductivity of a substrate doped with a small amount of normal saline or AuNPs colloidal solution we built tissue-mimicking phantoms based on agar gel (constituted by deionized water and 2 g/mL agar-agar powder). The phantoms had two compartments: a sphere mimicking a 4-cm doped zone, and a cylinder enclosing the sphere and mimicking non-doped tissue (see Fig. 5a). The sphere was located in the center of the cylinder.

While the cylinder was always made of agar gel, the spherical compartment was doped with one of two solutions: either 0.9% NaCl (Pisa Pharmaceutics, Guadalajara, Mexico) or 0.01 wt% colloidal AuNPs solution (10 nm average diameter) provided by the Physics Institute of UASLP (San Luis Potosí, Mexico). We assumed that these two dopant solutions did not occupy the entire doped area, but only a small percentage. In order to simulate this diffusion of the dopant in the medium, we considered two values for saline (1 and 1.5% wt) and one for colloidal gold (1% wt). For this we built four different spherical pieces to model four different conditions of the doped zone: (1) identical to the rest of the phantom (i.e., non-doped tissue, using a 33.5 cm^3^ agar gel volume); (2) spherical zone doped with 1 wt% of NaCl (using a mixture with 0.83 g of agar powder and 0.3 cm^3^ of 0.9% NaCl); (3) spherical zone doped with 1.5 wt% of NaCl (using a mixture with 0.8 g of agar powder and 0.5 cm^3^ of 0.9% NaCl); and (4) spherical zone doped with 1 wt% of AuNPs solution (using a mixture with 0.83 g of agar powder and 0.01 wt% AuNPs solution). For the doped sphere, the liquid components (deionized water ~ 80 °C and saline solution or AuNPs as appropriate) were mixed in a glass container together with the agar powder (2 g/mL). To ensure the homogeneity of the sample, the mixture was kept on a magnetic stirrer at constant temperature for 1 h. It was then poured into 4-cm-diameter spherical containers and kept at a temperature of 10 °C for 12 h to ensure solidification.

The cylindrical compartment was made in two stages. Agar powder (2 g/mL) was first mixed in deionized water at 80 °C, maintaining a constant temperature and stirring for 1 h. Subsequently, it was poured into a cylindrical container with an internal diameter of 10 cm and a height of 15 cm up to a third of its volume, allowing it to solidify at room temperature. Prior to complete solidification, the doped sphere was placed in the center of the cylinder and covered with the agar powder and deionized water mixture up to 11 cm in height. Once solidified, the entire phantom was kept in refrigeration at 10 °C at least 12 h before experiments.

**Fig. 6:**
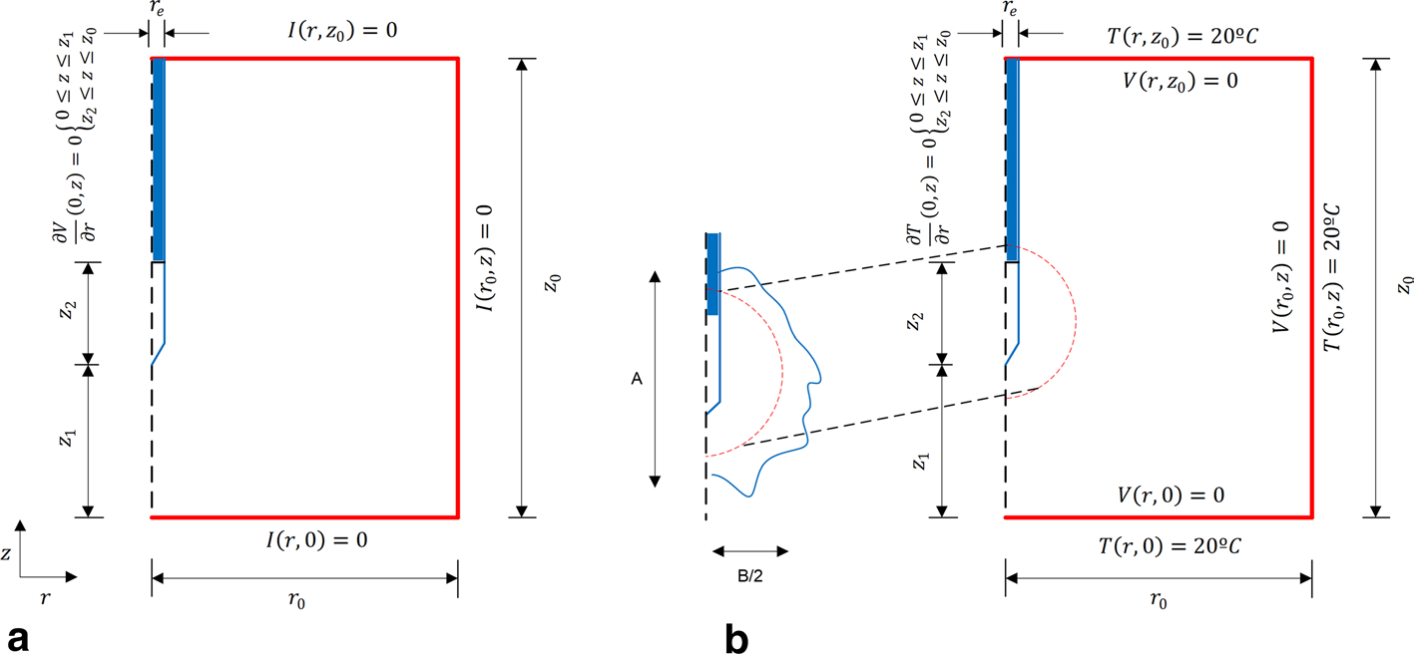
**a** Geometry and boundary conditions of the theoretical model used to estimate the values of electrical conductivity associated with the doped zone in the agar phantoms. Dimensions: *r*_0_ = 50 mm, *z*_0_ = 110 mm, *r*_i_ = 0.75 mm, *z*_1_ = 40 mm, *z*_2_ = 70 mm. *V*_i_ = 2 V. **b** 2D axisymmetric model used to study the temperature distributions during RF ablation of a doped zone with different dopants. It consisted of a cylinder of non-doped liver tissue (radius r_o_ = 10 cm and height *z*_0_ = 16 cm) surrounding a spherical doped zone of variable radius (dashed red line, *r*_t_ = 2, 3 and 4 cm). The active electrode (*r*_e_ = 0.75 mm) is inserted into the center of the doped zone. Solid blue line represents the contour of the coagulation zone, and A and B are the axial and transverse diameters, respectively

To estimate the electrical conductivity (*σ*) of the doped zone, we measured the impedance *Z* between a 3-cm active electrode model Cool-Tip (Medtronic, Minneapolis, MN, USA) inserted in the center of the spherical piece and a 2-mm-thick aluminum foil entirely surrounding the phantom and acting as a dispersive electrode (Fig. 5b). *Z* measurements were conducted by applying a sine voltage of 2 V amplitude and 500 kHz frequency. Voltage and current were measured by a digital oscilloscope TDS 3034B and a current probe mode A622, both from Tektronix (Beaverton, OR, USA). As expected, at RF frequencies the phantoms behaved electrically as pure resistors (no phase shift between current and voltage was observed), so that *Z* was inversely related to *σ*. Once Z measurements were obtained, we built a theoretical model and conducted computer simulations by changing *σ* values until we obtained the same *Z* values (i.e., a trial and error approach was used to estimate *σ*). The geometry, size and electrical boundary conditions of the theoretical model exactly mimicked the experimental conditions. Figure 6a shows the boundary conditions used. One-way analysis of variance was performed by the Fisher test (P = 0.05) to compare the *Z* values obtained with the four groups.

### Computer modeling of RFA in a doped zone

Computer modeling was used to study the effects of doping a tissue zone with NaCl and AuNPs on RFA electrical and thermal performance. Computer models were built and solved by finite element method using COMSOL Multiphysics software (Burlington, MA, USA). The problem represented a 2D axis symmetric model (see Fig. 6b) and consisted of a cylindrical domain mimicking non-doped liver tissue and a spherical domain mimicking a doped zone with variable diameter (2, 3 and 4 cm). This sensitivity analysis was motivated by the fact that the spatial distribution of the doping agent around the electrode was not really known. It also included an RF applicator identical to the Cool-Tip applicator used in the agar and ex vivo experiments. The dispersive electrode was modeled as an electrical boundary condition *V* = 0 on all the outer boundaries. The properties of the materials are shown in Table 7 [20]. The model was based on a coupled electric–thermal in which the governing equation for thermal problem was:

3 $$ \frac{\partial (\rho h)}{{\partial t}} = \nabla \cdot(k\nabla T) + q + Q_{p} , $$

where *ρ* (kg/m^3^) is tissue density, *h* (J/kg·K) enthalpy, *k* (W/m·K) thermal conductivity, *T* (°C) temperature, *t* (s) time, *q* the heat source and *Q*_*p*_ heat loss by blood perfusion (which is ignored since we are modeling ex vivo conditions). For biological tissues enthalpy is related to tissue temperature by the following expression [36]:

4 $$ \frac{\partial (\rho h)}{{\partial t}} = \frac{\partial T}{{\partial t}} \cdot \left\{ \begin{gathered} \rho_{l} c_{l\,} \quad 0 < T \le 99\;^\circ {\text{C}} \hfill \\ h_{fg} C\quad 99 < T \le 100\;^\circ {\text{C}} \hfill \\ \rho_{g} c_{g} \quad T > 100\;^\circ {\text{C}} \hfill \\ \end{gathered} \right., $$

where *ρ*_*i*_ and *c*_*i*_ are density and specific heat of tissue, respectively, at temperatures below 100 ºC (*i* = *l*) and at temperatures above 100 ºC (*i* = *g*), *h*_*fg*_ is the product of water latent heat of vaporization and water density at 100 ºC, and *C* is tissue water content inside the liver (68%) [37]. Equation (3) was applied to each region of the model by substitution of the appropriate properties. To calculate *Q*_*RF*_ we solved the electrical problem, which for RFA can be calculated by:

**Table 7:** Characteristics of the materials used in the computational model [20]

Material	*ρ* (kg/m^3^)	*σ* (S/m)	*c* (J/kg K)	*k* (W/m K)
Healthy tissue	1080	0.2	3455	0.502
Metal (electrode)	8000	7.4·10^6^	480	18
Plastic (insulated trocar)	70	10^−5^	1045	0.026

*ρ:* density, *σ:* electrical conductivity, *c*: specific heat, *k:* thermal conductivity. Tissue properties assessed at 37 °C

5 $${Q}_{\mathrm{RF}}={\varvec{J}}\bullet {\varvec{E}}=\sigma \cdot {\left|{\varvec{E}}\right|}^{2}=\boldsymbol{ }\sigma \cdot {\left|-\nabla V\right|}^{2},$$

where ***J*** is current density (A/m^2^), ***E*** electrical field (V/m), *σ* electrical conductivity (S/m) and *V* voltage (V). We used quasi-static approximation to solve the electromagnetic problem, where conduction currents were assumed to dominate compared to displacement currents. The electric voltage was computed by solving the equation [25]

6 $$\nabla \bullet (\sigma \nabla V)=0.$$

We assumed that the temperature dependence of electrical conductivity for both doped zone and for non-doped tissue was determined by:

7 $$ \sigma \left( T \right) = \sigma_{0} {\text{e}}^{{0.15\left( {T - T_{b} } \right)}} , $$

where sub-index $o$, indicates properties measured at *T*_*b*_ (37 °C). Damage to tissue is the effect of exposing it to a high temperature for a prolonged time. A traditional way to predict the probability of irreversible thermal damage is the Arrhenius reaction rate model:

8 $$ \Omega \left( t \right) = A\mathop \smallint \limits_{0}^{t} exp\left[ { - E_{a} /RT\left( \tau \right)} \right]d\tau , $$

where *Ω(t)* is the degree of tissue death, *A* is the frequency factor (7.39·10^39^ s^−1^), *E*_*a*_ is the activation energy for the irreversible damage reaction (2.557·10^5^ J/mol), and *R* is the universal gas constant (8.314 J/mol·K). The kinetic parameters (*A* and *E*_*a*_) that accounts for the morphological changes in tissue related to the thermal degradation of proteins were taken from [25]. A value *Ω* = 4.6 (which corresponds to 99% of cell death probability) was used to compute the coagulation zone boundary.

To determine how dopant solution within the tissue modifies the temperature distributions in the tissue during RFA, NaCl and AuNPs were assumed to change the properties of the tissue they came into contact with. The doped substrate was always assumed to coincide with the volume of a spherical zone around the electrode. To estimate the electrical conductivities for tissue doped with NaCl or AuNPs we used Eqs. (1) and (2), which were obtained from the results of the experiments on the agar model (see Results section), and where *σ*_S_ (electrical conductivity of the non-doped substrate) was that of the non-doped tissue (0.2 S/m in Table 7). Electrical impedance was assumed to increase by + 1.5%/°C until 100 °C. To model the tissue desiccation associated with the vaporization, when temperature reached 100 ºC we assumed that electrical conductivity dropped 2 orders of magnitude.

The other properties (density, volumetric heat specific and thermal conductivity) were estimated theoretically using the expression proposed in [34] for tissue characteristics according to the water content. Firstly, density of the doped tissue (*ρ*_DT_) can be determined as:

9 $$ \rho_{{{\text{DT}}}} = \left( {1 - \phi_{{\text{D}}} } \right)\rho + \phi_{{\text{D}}} \rho_{{\text{D}}} , $$

where *ρ* is the density of the non-doped tissue (see Table 7), *ρ*_D_ the density of dopant (NaCl solution or solid AuNPs (19,300 kg/m^3^)) and *Φ*_D_ denotes the volume fraction of dopant within the doped tissue. In the case of AuNPs, due to the extremely low value of *Φ*_D_ (e.g., ~ 50×10^−9^ in the case of 0.01 wt% of AuNPs occupying 1% by weight of doped tissue), *ρ*_DT_* ≈ ρ*, i.e., doped tissue density is hardly affected by the addition of the NPs. Likewise, in the case of NaCl solution, due to the similarity between its density (~ 1000 kg/m^3^) and that of the tissue (1080 kg/m^3^)), *ρ*_DT_* ≈ ρ*, i.e., the doped tissue density is little affected by the NaCl solution.

Volumetric specific heat of doped tissue (*ρc*)_DT_ can be similarly determined by:

10 $$ \left( {\rho c} \right)_{{{\text{DT}}}} = \left( {1 - \phi_{{\text{D}}} } \right)\left( {\rho c} \right) + \phi_{{\text{D}}} \left( {\rho c} \right)_{{\text{D}}} , $$

where *ρc* is the volumetric specific heat of the non-doped tissue, and *cρ*_D_ the volumetric specific heat of dopant. In the case of AuNPS, although the solid NPs have a much lower value (129 J/K·m^3^) than tissue (3455 J/kg·K), the extremely low value of *Φ*_D_ implies that the volumetric specific heat of the doped tissue is not greatly affected by the AuNPs. Likewise, due to the similarity between the volumetric specific heat of the NaCl solution (~ 4090 J/kg·K [38]) and the tissue, the NaCl solution has little effect on the volumetric specific heat of the doped tissue.

To determine the thermal conductivity in the doped tissue, we used the Maxwell–Eucken model [39] applied to suspension of particles:

11 $$ k_{DT} = k\cdot\frac{{k_{D} + 2k - 2\phi_{D} \left( {k - k_{D} } \right)}}{{k_{D} + 2k + \phi_{D} \left( {k - k_{D} } \right)}}, $$

where *k*_DT_ is a mixture of two phases, a continuous phase (the suspending liquid, non-doped tissue in our case) of conductivity *k* is the thermal conductivity of the non-doped tissue, and a disperse phase of dopant (NaCl solution or gold spherical NPs of conductivity *k*_D_ = 317 W/m K), and the volume fraction *Φ*_D_ of dispersal phase [40]. Once more, the extremely low value of *Φ*_D_ means that the thermal conductivity of the doped tissue is not much affected by the AuNPs, while the similarity between the thermal conductivity of the NaCl solution (~ 0.63 W/m·K [38]) and the tissue (0.502 W/m K) showed that that thermal conductivity of the doped tissue was not affected by the NaCl solution.

We assessed the effect doping the tissue with different concentrations *C* of 0.01% AuNPs colloidal solution, ranging from 0% (non-doped tissue) to 10%. Each concentration value was a value of a volume fraction of solid AuNPs in the doped tissue (*Φ*_D_). Table 6 summarizes the estimated characteristics of the doped tissue for different 0.9% NaCl solution and Au colloidal (0.01 wt%) concentration values distributed in the tissue (from 0 to 10%). Note that only the electrical characteristics were significantly modified by the dopants while the thermal properties remain unchanged.

To study the effects of the dopants on the coagulation zone created during RFA, we simulated three values of applied voltage: 50, 70 and 90 V. While the first (low voltage) is expected to avoid roll-offs for at least 10 min, the third (high voltage) is the standard value used in clinical practice for pulsed protocols [41]. All three values were expected to provide a preliminary insight into the effect of the dopant in terms of delaying roll-off, which was assumed to occur when impedance reached 100 Ω (initial impedance was always lower than this value). After computing the coagulation zone boundary by the *Ω* = 4.6 isoline, we computed the axial (A) and transverse (B) diameters (see Fig. 6b). Coagulation zone sphericity was assessed as A/B (values close to 1 are associated with spherical coagulation zones, while values greater than 1 are associated with ellipsoids).

### Ex vivo experimental setup

**Fig. 7:**
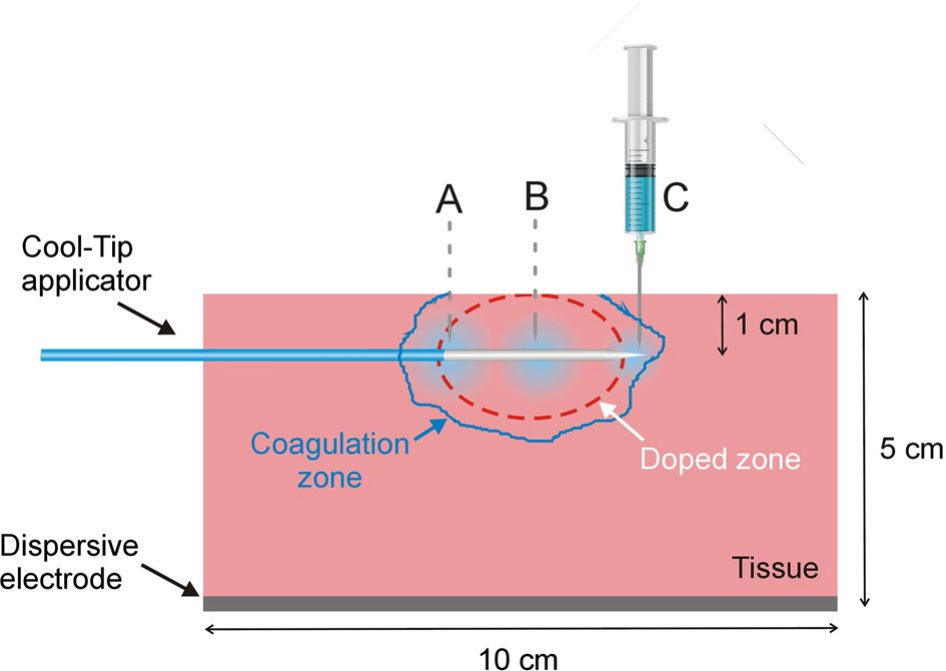
Cross-sectional view of tissue sample used in the ex vivo experiments. The ablation electrode was inserted 1 cm below the tissue surface. A, B, and C indicate the dopant infusion points (saline solution or Au colloidal). Solid blue line would represent the contour of the coagulation zone, while dashed red line would represent the contour of the doped zone

The experimental setup was based on an ex vivo model at room temperature (20 °C), which consisted of samples (8 × 10 × 5 cm ± 2 cm) of bovine liver acquired locally. The samples were placed on a metal plate which acted as a dispersive electrode. An active electrode model Cool-tip (Medtronic, Minneapolis, MN, USA) with 1.5 mm outside diameter and a 3-cm-long active tip was horizontally inserted ~ 1 cm into each sample (see Fig. 7). The electrode was internally cooled with circulating water (at 8 ± 2 °C) using a Masterflex L/S peristaltic pump (Cole-Parmer, Vernon Hills, IL, USA) at a rate of 40 mL/min. The pump was started at least 2 min before RFA to ensure effective cooling. Ablations were conducted by an RFG 3E-RF generator (Radionics, Burlington, MA, USA). Since the simulation results presented before suggested that highly conductive doped substrates possibly require moderate voltages, the RF generator set to ~ 57 V constant voltage was applied until roll-off (a variation of ± 2 V occurred between applications, due to the imprecision of the generator itself).

Three RFA protocols were tested: (1) non-doped tissue (ND Group), (2) previous infusion of 2 mL of 0.9% NaCl (NaCl Group), and (3) previous infusion of 2 mL of AuNPs 0.1 wt% (AuNPs Group). Note that a colloid with a higher concentration of AuNPs was used in the ex vivo experiments in case of higher differences than those suggested by the computer results. Each protocol was run on *n* = 4 samples. The infusion took around 1 min and was distributed at three points spaced 1 cm apart (~ 20 s each) located along the electrode length and at the same depth as the electrode. This was done to achieve a more or less homogeneous distribution of the dopant around the electrode (see Fig. 7) and was similar to the method used in [28]. RFA started immediately after infusion.

After completing each RFA, the ablation site in the tissue was identified by carefully cutting along the active electrode’s insertion path. The coagulation zone was defined *de visu* as the discolored white zone and images were taken with a digital camera. The axial and transverse diameters were measured by ImageJ software (National Institutes of Health, USA).

## Data Availability

All the data generated or analyzed during this study are included in this published article.
